# Supplementary material for: Two-dimensional ferroelasticity in van der Waals β’-In2Se3
Source: Nat Commun. 2021 Jun 16;12:3665. doi: 10.1038/s41467-021-23882-7 (PMC8209144; doi:10.1038/s41467-021-23882-7)
Supplement: Supplementary file 1 — Supplementary Information [file 41467_2021_23882_MOESM1_ESM.pdf]

Supplementary Information for

Two-dimensional ferroelasticity in van der Waals *β'*-In_2Se_3

Chao Xu[1], Jianfeng Mao[1], Xuyun Guo[1], Shanru Yan[1], Yancong Chen[2], Tsz Wing Lo[1], Changsheng Chen[1], Dangyuan Lei[3], Xin Luo[2], Jianhua Hao[1], Changxi Zheng[4,5] & Ye Zhu[1*]

[1]Department of Applied Physics, Research Institute for Smart Energy, The Hong Kong Polytechnic University, Hung Hom, Kowloon, Hong Kong, China
[2]State Key Laboratory of Optoelectronic Materials and Technologies, Centre for Physical Mechanics and Biophysics, School of Physics, Sun Yat-sen University, Guangzhou 510275, China
[3]Department of Materials Science and Engineering, City University of Hong Kong, 83 Tat Chee Avenue, Hong Kong, China
[4]School of Science, Westlake University, Hangzhou 310024, China
[5]Institute of Natural Sciences, Westlake Institute for Advanced Study, Hangzhou 310024, China

[*]Email: yezhu@polyu.edu.hk

Outline

I. Supplementary Figures

1. **Supplementary Figure 1.** Powder X-ray diffraction (PXRD) of β'-In_2Se_3.
2. **Supplementary Figure 2.** Strain calibration using strain gauge.
3. **Supplementary Figure 3.** Raman spectroscopy and second harmonic generation (SHG) characterization on In_2Se_3.
4. **Supplementary Figure 4.** AFM nano-indentation on CVD-grown β'-In_2Se_3.
5. **Supplementary Figure 5.** Ferroelastic domain switching involving all three domain variants in an exfoliated β'-In_2Se_3 flake.
6. **Supplementary Figure 6.** Domain structure observed in a 6-layer-thick β'-In_2Se_3 flake.
7. **Supplementary Figure 7.** Domain structure in exfoliated 3R β'-In_2Se_3, showing high priority of 60° DWs.
8. **Supplementary Figure 8.** Ferroelastic strain caused in-plane (IP) and out-of-plane (OOP) deviation angles in β'-In_2Se_3.
9. **Supplementary Figure 9.** PFM measurements on typical ferroelectrics and non-ferroelectrics.
10. **Supplementary Figure 10.** Lateral PFM on a ferroelectric PZT thin film and a non-ferroelectric 3R β'-In_2Se_3 flake.
11. **Supplementary Figure 11.** DWs across a step edge and the corresponding lateral PFM images in 2H β'-In_2Se_3.

II. Supplementary Notes

1. **Supplementary Note 1:** Permissible ferroelastic domain walls (DWs) in 2D β'-In_2Se_3.
2. **Supplementary Note 2:** Transformation strain between domain variants.
3. **Supplementary Note 3:** Breaking strength and strain measured by AFM nano-indentation.
4. **Supplementary Note 4:** Ferroelastic species and 3D spontaneous strain in β'-In_2Se_3.
5. **Supplementary Note 5:** Origin of the PFM domain contrast in 3R β'-In_2Se_3.

III. Supplementary Tables

1. **Supplementary Table 1.** Ferroelastic species of 2H and 3R β'-In_2Se_3.
2. **Supplementary Table 2.** Permissible DWs and their 3D orientations in β'-In_2Se_3.

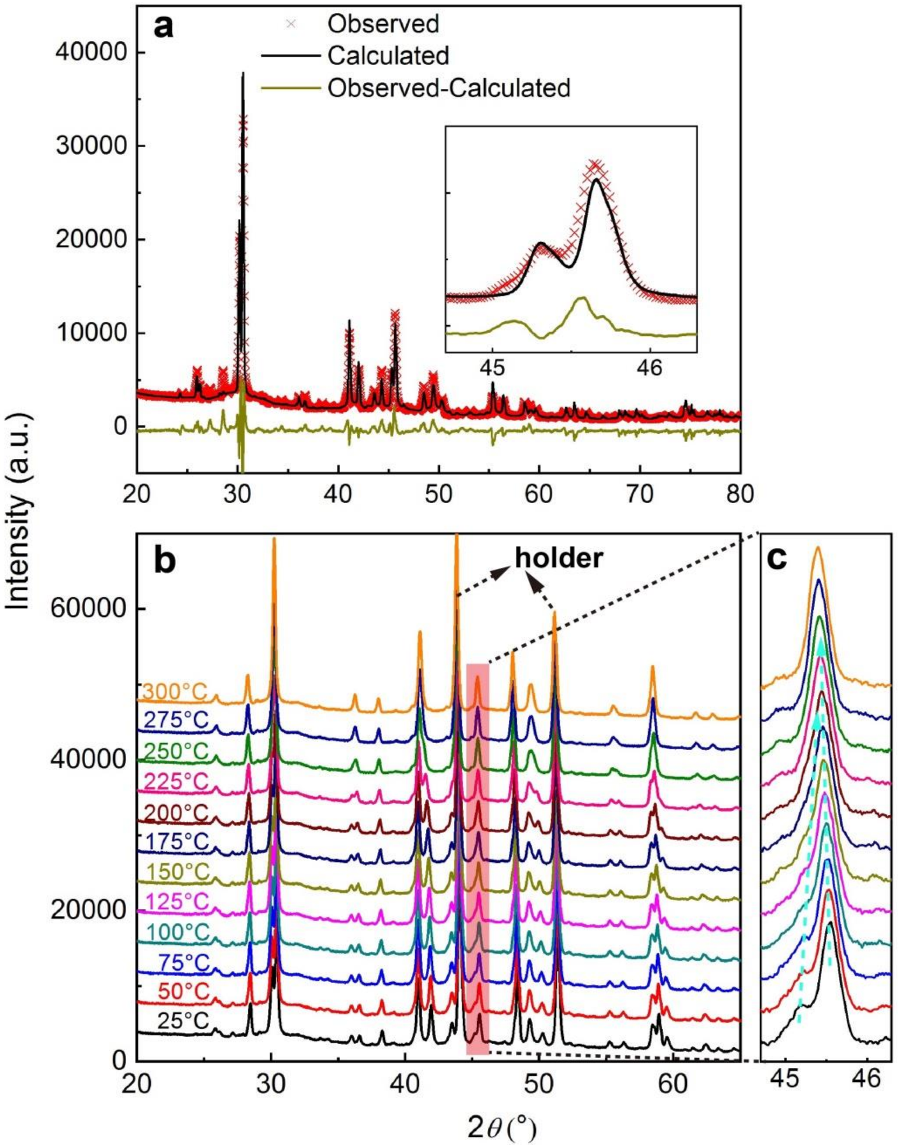

Supplementary Figure 1. Powder X-ray diffraction (PXRD) of *β'*-In_2Se_3. a PXRD at room temperature and the calculated pattern based on Rietveld refinement using the first-principles predicted structure as the input. The inset manifests the $11\bar{2}0$ peak splitting at ~45.5° due to the in-plane lattice distortion that breaks symmetry. The intensity of the left peak at ~45.3° is approximately half of the right peak (~45.6°), consistent with the lattice distortion model in Fig. 1d: the lattice dilation parallel to nanostripes/polarization direction enlarges $d^{\parallel}_{11\bar{2}0}$ (the left peak), while the lattice compression perpendicular to nanostripes reduces the spacing of the other two $\{11\bar{2}0\}$ planes with ±120° with respect to nanostripes (the right peak). **b** *In situ* PXRD upon heating to 300°C. The diffraction signal from the *in situ* holder itself is indicated by the black dashed arrows. **c** Highlight showing the evolution of the $11\bar{2}0$ peak upon increasing temperature (cyan arrows).

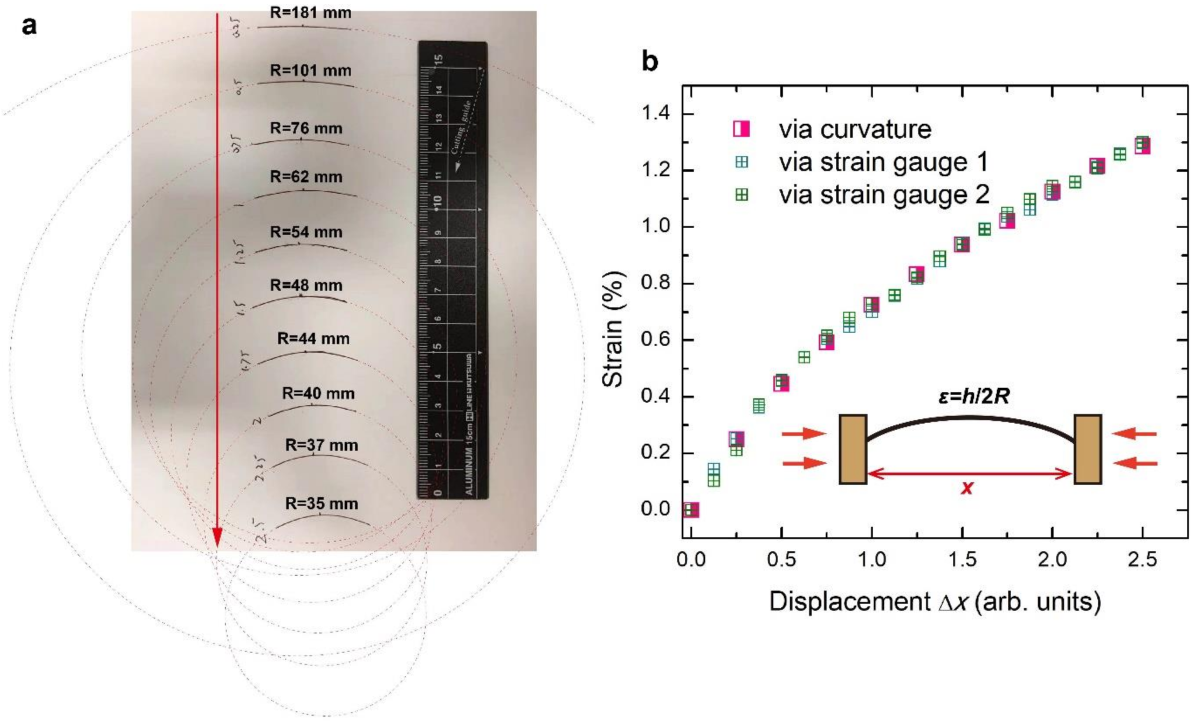

Supplementary Figure 2. Strain calibration using strain gauge. a Radii of curvature (R) measurement in the two-point bending experiment. **b** Strain values obtained from the substrate radii of curvature and the two strain gauges attached on the substrates. The lower inset in **b** shows the geometry of the two-point bending setup. The strain in each step is controlled by setting the relative displacement Δx of the two terminals.

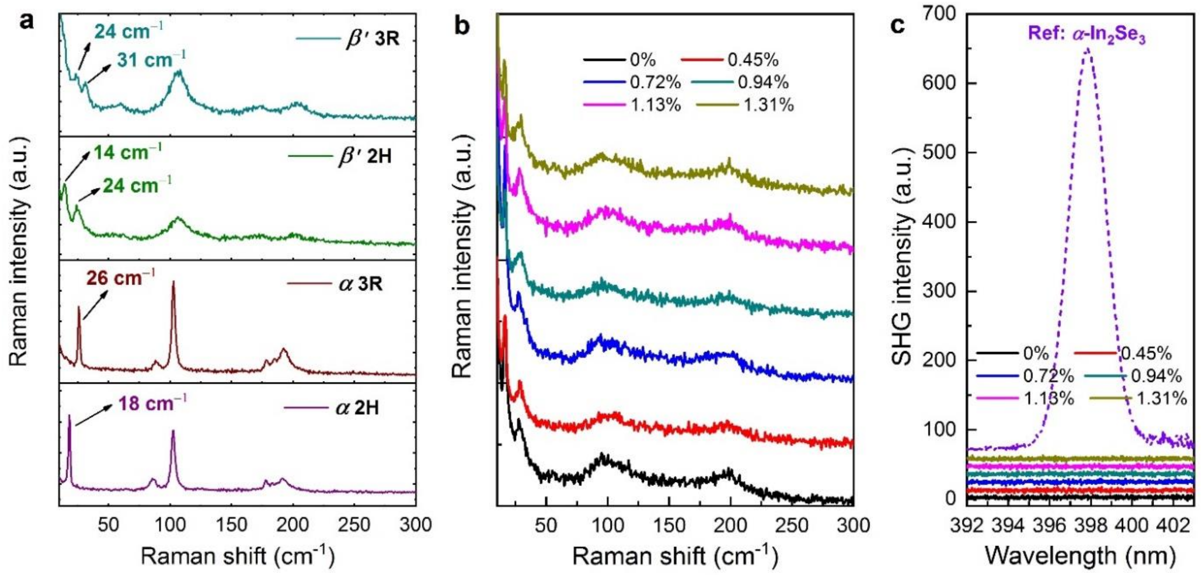

Supplementary Figure 3. Raman spectroscopy and second harmonic generation (SHG) characterization on In_2Se_3. a Raman spectra for α-/β'-In_2Se_3 with 2H/3R stacking with the low-frequency Raman shifts labelled in each spectrum. Raman peaks near 86, 102, 178, 185, and 193 cm^{-1} are observed in 2H/3R α-In_2Se_3, and near 60, 106, 173, and 202 cm^{-1} are observed in 2H/3R β'-In_2Se_3 flakes, consistent with previous studies[1,2]. These peaks are merely related to the intralayer structure and independent on the stacking order, and thus can be used to distinguish α- and β'-In_2Se_3. In addition to the distinct peak positions, β'-In_2Se_3 also shows broader Raman peaks than α-In_2Se_3 due to the strong vibrational anharmonicity[3]. In the low-frequency range from 10 to 50 cm^{-1}, there are peaks at 18 and 26 cm^{-1} for 2H and 3R α-In_2Se_3 respectively, and peaks at 14 and 24 cm^{-1} for 2H and 24 and 31 cm^{-1} for 3R β'-In_2Se_3. These Raman peaks are presumably related to the interlayer vibrational modes, and can thus be used to distinguish the interlayer stacking order.

b *In situ* Raman spectroscopy and **c** SHG from tensile-strained β'-In_2Se_3. An α-In_2Se_3 flake is taken for reference in the SHG measurement as there is no SHG response in β'-In_2Se_3. Raman spectroscopy was carried out on a Witec confocal microscopy with a 532 nm exciting laser. SHG was collected with a monochromator (Princeton SpectraPro 2750 integrated with a ProEM EMCCD camera with a spectral resolution less than 0.1 nm) under a Ti:sapphire femtosecond laser source (Coherent Libra) centered at 800 nm.

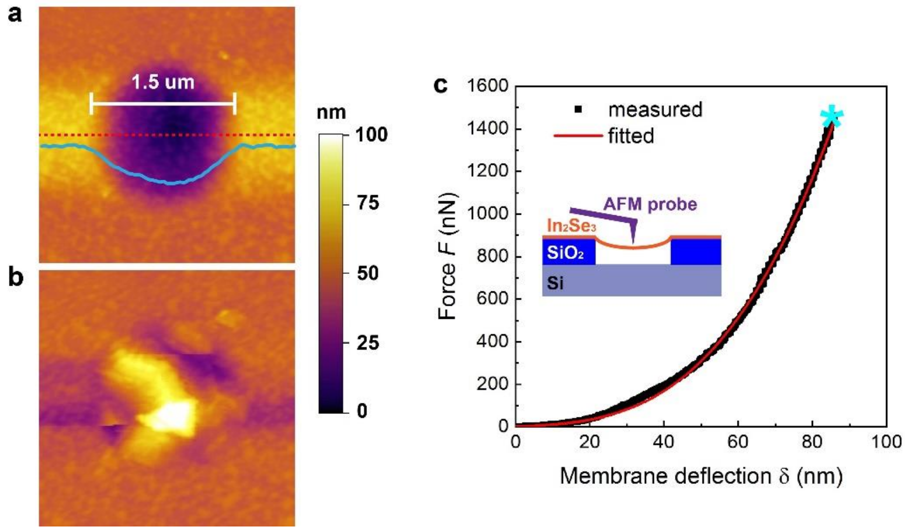

Supplementary Figure 4. AFM nano-indentation on CVD-grown *β'*-In_2Se_3 transferred onto holey SiO_2@Si substrates. a AFM image of a ~14 nm thin In_2Se_3 flake suspended over a 1.5 μm hole for the indentation experiment. The profile in blue across the holey region (red dashed line) suggests pre-strain of the suspended area. **b** Fracture of the thin In_2Se_3 flake after large-force loading. **c** The typical loading curves for a *β'*-In_2Se_3 flake and the least-squares fit. The fracture point is marked by the symbol *. The inset shows the schematic of the indentation experiment on the suspended In_2Se_3 flake.

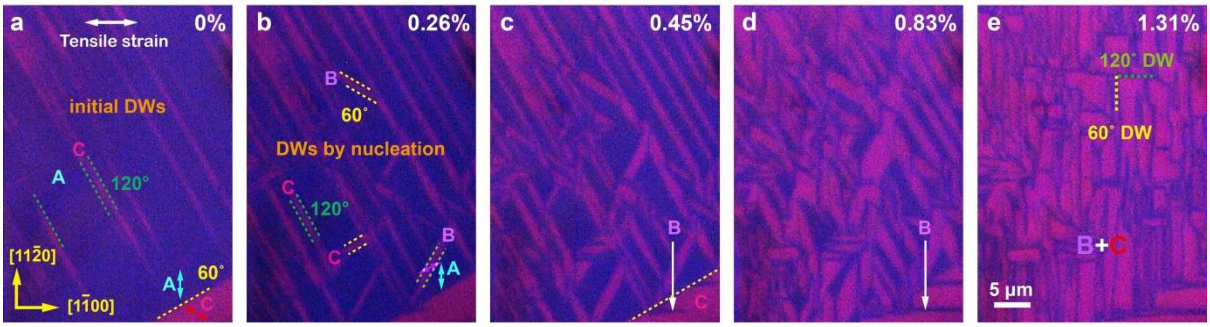

Supplementary Figure 5. Ferroelastic domain switching involving all three domain variants in an exfoliated *β'*-In_2Se_3 flake. a Polarized light image showing the initial 60° and 120° DWs. **b** Formation of new 60° and 120° DWs by domain nucleation at the first stage of the switching process under the horizontal tensile strain. **c** Nucleation of B domains inside a C domain in the lower-right corner, as indicated by the white arrow. **d** Complex domain switching between B and C variants. **e** Presence of orthogonal 60° and 120° DWs at the final stage when most A variant switches to B and C variants.

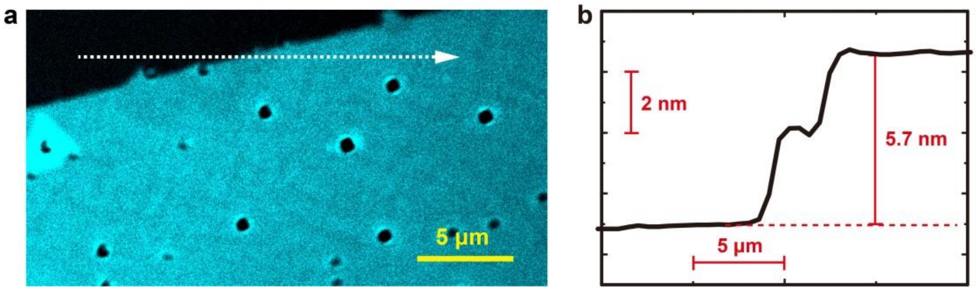

Supplementary Figure 6. Multidomain pattern observed in a 6-layer-thick *β*'-In_2Se_3 flake. **a** Polarized light image showing multidomain contrast. The dark patches are contaminants at the flake surface. **b** Height profile along the white dashed arrow in **a**, indicating the flake thickness of ~5.7 nm.

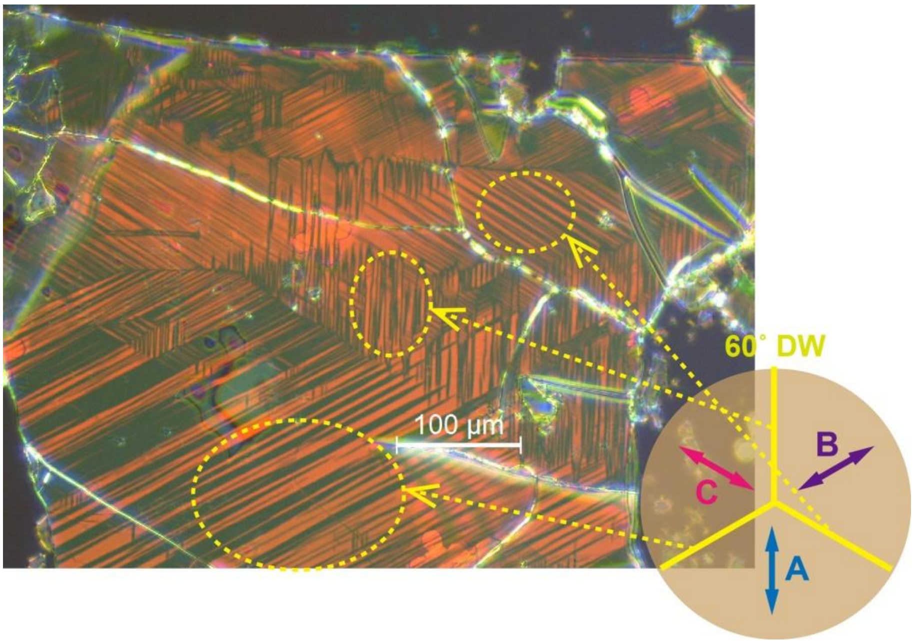

Supplementary Figure 7. Multidomain pattern in exfoliated 3R *β*'-In_2Se_3, showing the predominance of 60° DWs with few 120° DWs. The lower right schematic explains the associated ferroelastic domain variants with lattice dilation directions indicated by double-headed arrows (same as the bottom inset in Fig. 3a).

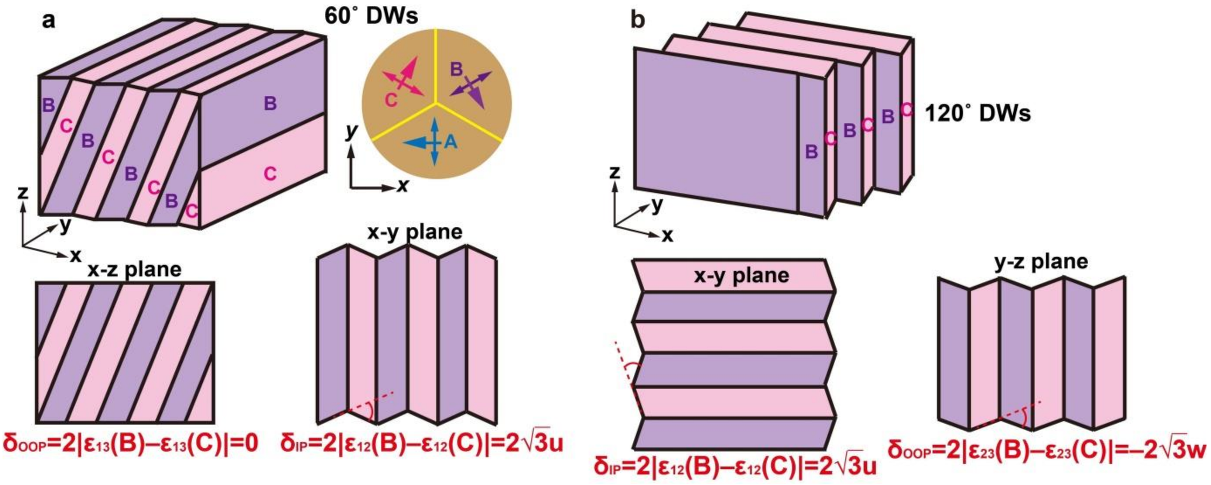

Supplementary Figure 8. In-plane (IP) and out-of-plane (OOP) deviation angles in 60° (a) and 120° (b) DWs, caused by 3D ferroelastic strain in 3R *β*'-In_2Se_3. For both 60° and 120° DWs in 3R *β*'-In_2Se_3, the IP (*x*-*y* plane) deviation angle δ_{IP}(60°/120°) = $2|\varepsilon_{12}(\mathrm{B})-\varepsilon_{12}(\mathrm{C})| = 2\sqrt{3}u = 0.017$ rad $= 0.97°$ (see Supplementary Note 4). As for the OOP deviation angles, δ_{OOP}(60°) = $2|\varepsilon_{13}(\mathrm{B})-\varepsilon_{13}(\mathrm{C})|$ = 0 (components ε_{13} in B and C variants sharing the same sign, see the top right inset) and δ_{OOP}(120°) = $2|\varepsilon_{12}(\mathrm{B})-\varepsilon_{12}(\mathrm{C})| = -2\sqrt{3}w = 0.0436$ rad $= 2.50°$ (see Supplementary Note 4). The non-zero OOP deviation angle gives rise to surface wrinkles across 120° DWs in 3R *β*'-In_2Se_3, while the zero OOP deviation angle indicates flat surface across 60° DWs.

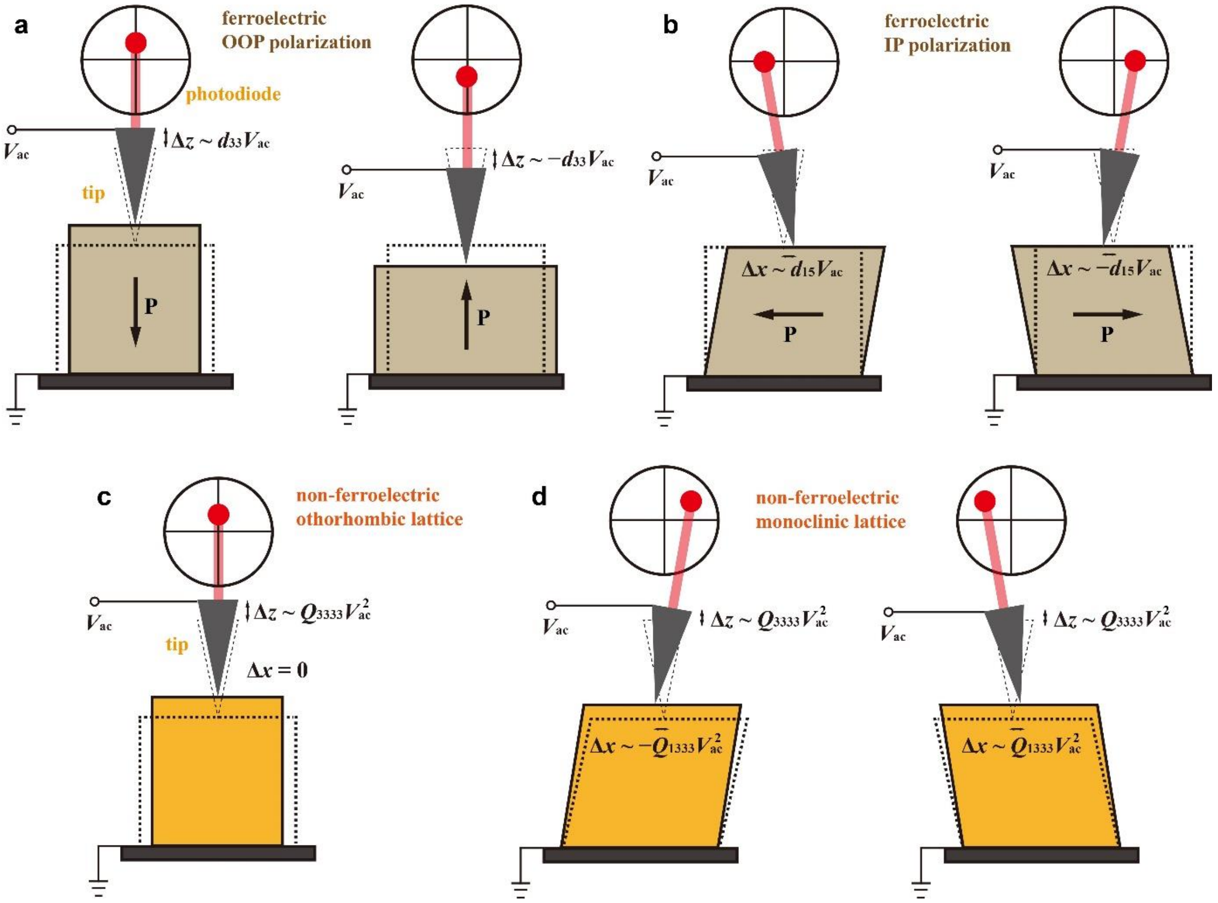

Supplementary Figure 9. PFM measurements on typical ferroelectrics and non-ferroelectrics. a, b Piezoelectric response characterized by PFM with OOP polarization (a) and IP polarization (b). **c, d** Piezoelectric response characterized by PFM in non-ferroelectrics with orthorhombic lattice (c) and monoclinic lattice (d). See Supplementary Note 5 for detailed discussion.

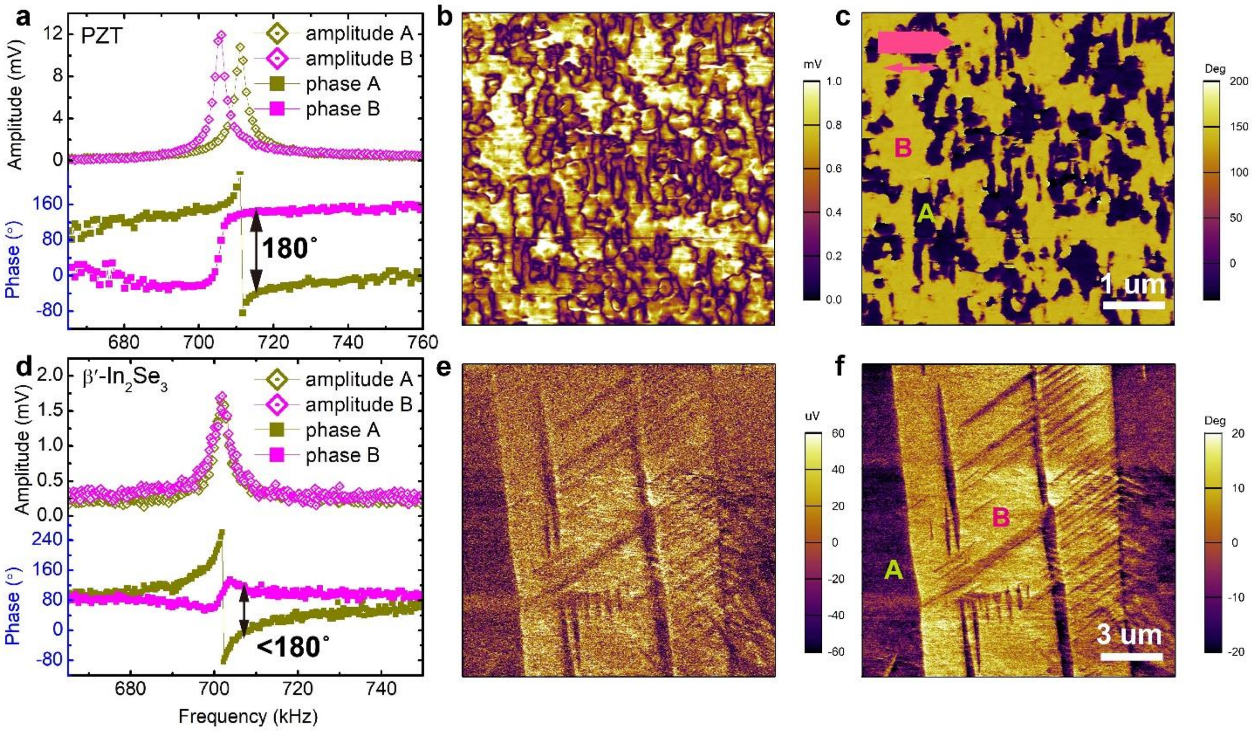

Supplementary Figure 10. Lateral PFM on a ferroelectric lead zirconate titanate (PZT) thin film and a non-ferroelectric 3R β'-In_2Se_3 flake. **a, d** In-plane amplitude and phase frequency responses of two different domains in a PZT thin film (a) and a 3R β'-In_2Se_3 flake (d) across the resonance. **b, c** Amplitude and **c, f** phase mapping of the PZT thin film (b, c) and 3R β'-In_2Se_3 flake (e, f) under a driven frequency slightly above the resonance. The less than 180° phase difference both across the resonance and between the domains in 3R β'-In_2Se_3 indicates its non-ferroelectric nature in contrast to PZT. See Supplementary Note 5 for detailed discussion.

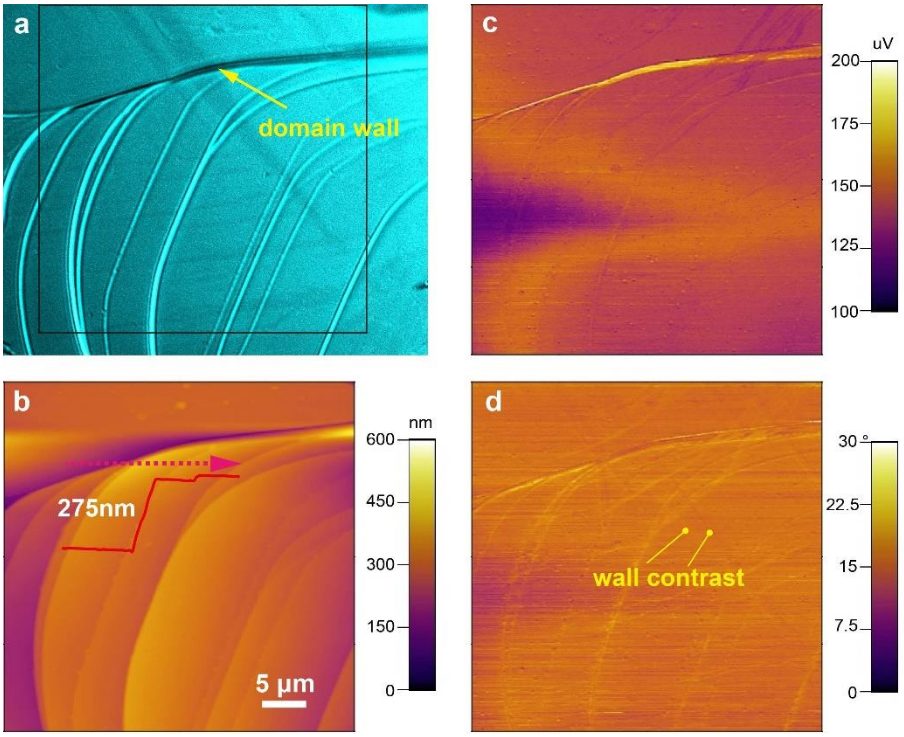

Supplementary Figure 11. DWs across a step edge and the corresponding lateral PFM images in 2H β’-In_2Se_3. a Polarized light image showing no horizontal shift of DWs across the step edge, reflecting the vertical nature in contrast to the inclined DWs in 3R β’-In_2Se_3. **b-d** AFM topography (b), lateral PFM amplitude (c) and phase (d) images of the area highlighted by the black box in **a**. No domain contrast is observed in lateral PFM images, further confirming the non-ferroelectric nature of these ferroelastic domains.

Supplementary Note 1: Permissible ferroelastic domain walls in 2D β'-In_2Se_3.

With the 2D spontaneous strain in antiferroelectric β'-In_2Se_3, the orientation of a mechanically compatible domain wall (DW) with the normal vector **n** = [x_1, x_2] can be determined from the equation for mechanically compatible interfaces[4,5]

$$\sum_{m,n=1}^{2}[\varepsilon_{mn}(+\infty) - \varepsilon_{mn}(-\infty)]x_m x_n = 0, \quad (1)$$

where ε_{mn} (m, n=1, 2) is the 2D spontaneous strain component. Substituting $\boldsymbol{\varepsilon}(+\infty)$ and $\boldsymbol{\varepsilon}(-\infty)$ with the strain tensors of different domain variants, all permissible DWs can be derived. Considering the domain configuration as depicted in Fig. 3a and setting $\boldsymbol{\varepsilon}(+\infty) = \boldsymbol{\varepsilon}(\mathrm{B})$ and $\boldsymbol{\varepsilon}(-\infty) = \boldsymbol{\varepsilon}(\mathrm{C})$, we have

$$[\varepsilon_{11}(\mathrm{B}) - \varepsilon_{11}(\mathrm{C})]x^2 + [\varepsilon_{12}(\mathrm{B}) - \varepsilon_{12}(\mathrm{C})]xy + [\varepsilon_{21}(\mathrm{B}) - \varepsilon_{21}(\mathrm{C})]xy + [\varepsilon_{22}(\mathrm{B}) - \varepsilon_{22}(\mathrm{C})]y^2 = 0. \quad (2)$$

The above equation can be reduced to

$$xy = 0, \quad (3)$$

with the two solutions

$$x = 0 \text{ and } y = 0. \quad (4)$$

As x and y are defined as $[1\bar{1}00]$ and $[11\bar{2}0]$ directions, respectively, x = 0 corresponds to the DW perpendicular to the $[1\bar{1}00]$ direction or parallel to the $(1\bar{1}00)$ plane, thus the 60° DW as shown in Fig. 3a. y = 0 corresponds to the 120° DW that is perpendicular to the $[11\bar{2}0]$ direction or parallel to the $(11\bar{2}0)$ plane. With DWs between all domain variant pairs solved likewise, the 60° DWs always lie along $\{1\bar{1}00\}$ planes while the 120° DWs always along $\{11\bar{2}0\}$ planes, as illustrated by yellow and green lines in Fig. 3a.

Supplementary Note 2: Transformation strain between domain variants.

As ferroelastic domains can be switched upon external strain, the associated transformation strain is given by the strain difference between the domain pairs. For the switching process described in Fig. 4, the transformation strain is

$$\boldsymbol{\varepsilon}_{\mathrm{B}}^{\mathrm{A}} = \boldsymbol{\varepsilon}_{\mathrm{A}} - \boldsymbol{\varepsilon}_{\mathrm{B}} = \begin{bmatrix} -0.0074 & -0.0042 \\ -0.0042 & 0.0074 \end{bmatrix}. \quad (5)$$

Thus the domain switching from B to A variant is associated with 0.74% elongation/ compression along $[11\bar{2}0]$ / $[1\bar{1}00]$ directions. With the experimental setup in Fig. 4,

assuming all the applied tensile strain ε_{ext} is accommodated by domain switching, and also neglecting the transverse constraint of the substrate – the so-called freestanding switching model, we have $\varepsilon_{ext} = 0.74\% \times \Delta F$, where ΔF represents the domain switching fraction. This linear dependence of ΔF on ε_{ext} can be plotted as the blue line in Fig. 4h with a slope value 1/0.74 = 1.35, which gives a good description to the experimental data.

Supplementary Note 3: Breaking strength and strain measured by AFM nano-indentation.

The AFM nano-indentation is a common method for studying the mechanical properties of 2D materials[6-9]. Here we conducted AFM indentation test on a CVD-grown flake of ~14 nm thick to estimate the breaking strength of vdW β'-In_2Se_3. As shown in Supplementary Fig. 4, the membrane is suspended over a circular hole with radius r = 750 nm and deformed in the center by an AFM tip (with a radius r_{tip} < 25 nm).

With $r_{tip} \ll r$, the classical theory of continuum mechanics can be applied[7]. The deformation behavior of a suspended 2D flake during indentation can be approximated by the equation[10]:

$$F = \sigma_0^{2D} \pi \delta + E^{2D} \frac{q^3 \delta^3}{r^2}. \quad (6)$$

The first term linear with deflection δ corresponds to the linear pre-stretched membrane regime with σ_0^{2D} as the prestress in the membrane. The second term represents the modified form of the classical Schwerin solution for point loading on the circular suspended thin membrane and is related to the elastic modulus E^{2D}. The dimensionless constant q depends on the Poisson ratio v as $q = 1/(1.05 - 0.15v - 0.16v^2)$. The Poisson ratio of $v \sim 0.25$ for vdW In_2Se_3 gives q=0.998[11].

By fitting the measured force-deflection data (Supplementary Fig. 4c) using the above formula, the elastic modulus E^{2D} of the β'-In_2Se_3 2D flake can be extracted to be 1429 ± 178 N/m. With the derived elastic modulus, the maximum stress σ_{max}^{2D} can then be calculated to be 80 ± 4 N/m using the expression for the indentation of a clamped, linearly elastic membrane by a small spherical indenter ($r_{tip}/r \ll 1$, with r_{tip} = 25 nm and r = 750 nm) at its loading limit:

$$\sigma_{max}^{2D} = \sqrt{\frac{F_{max} E^{2D}}{4 \pi r_{tip}}}. \quad (7)$$

Considering the flake thickness of ~14 nm, the effective Young's modulus $E^{eff} = 102 \pm 13$

GPa and breaking strength σ_{max}^{eff} = 5.7 ± 0.3 GPa can be obtained. These values closely match the predicted ones for monolyer β-In_2Se_3[12], which validates our nano-indentation measurement. A linear approximation of the mechanical relationship between the stress and strain for stiff materials further gives the breaking or yield strain ~5.5% ($\sigma_{max}^{eff}/E^{eff}$)[9], much larger than the strain required for ferroelastic domain switching.

Supplementary Note 4: Ferroelastic species and 3D spontaneous strain in β'-In_2Se_3.

The 2H (AB'AB') and 3R (ABCABC) stacking of the β'-In_2Se_3 quintuple layers result in two distinct ferroelastic species - $6mm$–$mm2$ and $\bar{3}m$–$2/m$ (see Supplementary Table 1), both give triply degenerated domain states[13,14]. Following the domain configuration depicted in Fig. 3a, the Aizu spontaneous strain of the A variant in 2H and 3R β'-In_2Se_3 can be described respectively as

$$\boldsymbol{\varepsilon}(\mathrm{A, 2H}) = \begin{pmatrix} -u & 0 & 0 \\ 0 & u & 0 \\ 0 & 0 & 0 \end{pmatrix}, \quad (8)$$

$$\boldsymbol{\varepsilon}(\mathrm{A, 3R}) = \begin{pmatrix} -u & 0 & w \\ 0 & u & 0 \\ w & 0 & 0 \end{pmatrix}, \quad (9)$$

while strain of the B and C variants are in-plane rotated by φ = 120° and –120° and thus can be obtained via 3D matrix transformation

$$\boldsymbol{\varepsilon} = \mathbf{J}\boldsymbol{\varepsilon}(\mathrm{A})\mathbf{J}^{-1}, \mathbf{J} = \begin{pmatrix} \cos\varphi & -\sin\varphi & 0 \\ \sin\varphi & \cos\varphi & 0 \\ 0 & 0 & 1 \end{pmatrix}. \quad (10)$$

The transformation gives the strain tensors as

$$\boldsymbol{\varepsilon}(\mathrm{B, 2H}) = \begin{pmatrix} u/2 & \sqrt{3}u/2 & 0 \\ \sqrt{3}u/2 & -u/2 & 0 \\ 0 & 0 & 0 \end{pmatrix}, \quad (11)$$

$$\boldsymbol{\varepsilon}(\mathrm{C, 2H}) = \begin{pmatrix} u/2 & -\sqrt{3}u/2 & 0 \\ -\sqrt{3}u/2 & -u/2 & 0 \\ 0 & 0 & 0 \end{pmatrix}, \quad (12)$$

$$\varepsilon(\mathrm{B},3\mathrm{R}) = \begin{pmatrix} u/2 & \sqrt{3}u/2 & -w/2 \\ \sqrt{3}u/2 & -u/2 & \sqrt{3}w/2 \\ -w/2 & \sqrt{3}w/2 & 0 \end{pmatrix}, \quad (13)$$

$$\varepsilon(\mathrm{C},3\mathrm{R}) = \begin{pmatrix} u/2 & -\sqrt{3}u/2 & -w/2 \\ -\sqrt{3}u/2 & -u/2 & -\sqrt{3}w/2 \\ -w/2 & -\sqrt{3}w/2 & 0 \end{pmatrix}. \quad (14)$$

The strain component *u* and *w* can be derived from PXRD shown in Supplementary Fig. 1 and at room temperature the values of *u* and *w* are 0.0049 and –0.0126, respectively. The 3D orientations of the DWs can be solved as well based on the mechanical compatibility criterion (see Note 1), as listed in Supplementary Table 2. The 120° DWs in both 2H and 3R *β*'-In_2Se_3 are fixed with regard to the mirror plane $\{11\bar{2}0\}$ of the prototypic *β* phase and are called W_f-type walls, while the 60° DWs depend on the spontaneous strain (*u* and *w* herein) and are called *S*-type walls[5].

Supplementary Note 5: Origin of the PFM domain contrast in 3R *β'*-In_2Se_3.

In typical ferroelectrics, the first-order electrostrictive effect (piezoelectricity) is strong and dominates the PFM response. As illustrated in Supplementary Figs. 9a and 9b, under a modulated cantilever voltage $V_{ac} = V_0 \cos \omega t$, the out-of-plane (OOP) and in-plane (IP) piezoresponses can be described as

$$\Delta z = d_{33} V_{ac}, \quad (15)$$

$$\Delta x = d_{15} V_{ac}, \quad (16)$$

where d_{33} and d_{15} are the longitudinal and shear piezocoefficients, respectively. As demonstrated by Sader[15], in the vicinity of a resonance with small damping (quality factor $Q > 10$), the amplitude and phase frequency responses can be described using the harmonic oscillator model as

$$A(\omega) = \frac{A_{max}\omega_0^2/Q}{\sqrt{\left(\omega_0^2-\omega^2\right)^2+(\omega_0\omega/Q)^2}}, \quad (17)$$

$$\tan\varphi(\omega) = \frac{\omega_0\omega}{Q(\omega_0^2-\omega^2)}. \quad (18)$$

Considering two oppositely oriented ferroelectric domains with IP polarization, for instance, the PFM responses are opposite in sign

$$\Delta x_{+}(\omega) = d_{15}V_{ac} = d_{15}V_0 \cos\omega t, \qquad (19)$$

$$\Delta x_{-}(\omega) = -d_{15}V_{ac} = d_{15}V_0 \cos(\omega t + \pi). \qquad (20)$$

Thus in ferroelectrics, according to the above equations, there is a phase jump of 180° across the resonant point and a phase difference of 180° off the resonance between oppositely oriented domains[16].

On the other hand, non-ferroelectric dielectrics can also show electromechanical response through the second-order electrostrictive effect, which is quadratic in the applied voltage[17,18], as shown in Supplementary Figs. 9c and 9d. Thus the OOP and IP PFM response are

$$\Delta z = Q_{3333}(\varepsilon_{33}V_{ac})^2 = Q_{3333}\, V_{ac}^2 \cos^2\omega t = Q_{3333}V_{ac}^2(\cos 2\omega t + 1)/2, \quad (21)$$

$$\Delta x = Q_{1333}(\varepsilon_{33}V_{ac})^2 = Q_{1333}\, V_{ac}^2 \cos^2\omega t = Q_{1333}V_{ac}^2(\cos 2\omega t + 1)/2. \quad (22)$$

These equations suggest a second-harmonic response with the driving force resulted from the second-order electrostrictive effect, which is significantly different from the piezoelectricity. Considering oppositely oriented domains with IP shear strain, the second-harmonic responses are

$$\Delta x_{+}(\omega) = Q_{1333}V_{ac}^2(\cos 2\omega t + 1)/2, \qquad (23)$$

$$\Delta x_{-}(\omega) = -\,Q_{1333}V_{ac}^2(\cos 2\omega t + 1)/2. \qquad (24)$$

Thus as long as Q_{1333} is non-zero (non-zero IP shear strain), the domains can still be imaged. But the phase change from this nonlinear response will be ~90° for ω instead of 180°. On the other hand, the current PFM instrument is not designed to work on such second-order electrostrictive effect, thus the measured the phase difference may deviate from 90°, but definitely much less than 180°.

To further illustrate the non-ferroelectric origin of the PFM contrast, Supplementary Fig. 10 compares the lateral PFM response of the 3R β'-In_2Se_3 flake with the ferroelectric lead zirconate titanate (PZT) thin film. As expected, the amplitude/phase frequency responses as well as the lateral PFM imaging between the two materials behave differently. The PZT thin film exhibits typical ferroelectric behavior – a phase change of 180° across the resonant point and a phase difference of 180° between domains, while the 3R β'-In_2Se_3 flake shows smaller phase difference that are consistent with the above second-order electrostrictive effect for non-

ferroelectrics. Furthermore, for 2H β'-In_2Se_3, because of the zero in-plane electromechanical response ($Q_{1333} = 0$) of the orthorhombic lattice (Supplementary Fig. 9c), consequently there's negligible contrast over the ferroelastic domains, as shown in Supplementary Fig. 11, providing another evidence that the domains in β'-In_2Se_3 are not ferroelectric. Therefore, we conclude that the domain contrast in 3R β'-In_2Se_3 arises from the second-order electrostrictive effect rather than ferroelectricity.

Supplementary Table 1. Ferroelastic species of 2H and 3R *β*'-In_2Se_3.

Stacking order	Parent *β* phase		Ferroelastic *β*' phase	
	Space group	Point group	Space group	Point group
2H	$P63mc$	$6mm$	$Pca2_1$	$mm2$
3R	$R\bar{3}m$	$\bar{3}m$	$P2/c$	$2/m$

Supplementary Table 2. Permissible DWs and their 3D orientations in *β*'-In_2Se_3.

Domain pair	DWs in 2H *β*'-In_2Se_3		DWs in 3R *β*'-In_2Se_3	
	120° (W_f type)	60° (S type)	120° (W_f type)	60° (S type)
B-C	$y = 0$	$x = 0$	$y = 0$	$z = -u/wx$*
A-B	$y = \sqrt{3}x$	$x = -\sqrt{3}y$	$y = \sqrt{3}x$	$u(\sqrt{3}y + x) - 2wz = 0$
A-C	$y = -\sqrt{3}x$	$x = \sqrt{3}y$	$y = -\sqrt{3}x$	$u(-\sqrt{3}y + x) - 2wz = 0$

* The non-zero *u* and *w* give rise to inclined 60° DWs in 3R *β*'-In_2Se_3.

References

1. Tao, X. & Gu, Y. Crystalline-crystalline phase transformation in two-dimensional In_2Se_3 thin layers. *Nano Lett.* **13**, 3501-3505, (2013).
2. Liu, L. *et al.* Atomically resolving polymorphs and crystal structures of In_2Se_3. *Chem. Mater.* **31**, 10143-10149, (2019).
3. Igo, J. *et al.* Anharmonic phonon coupling in single-crystal semiconducting and metal-like van der Waals In_2Se_3. *J. Phys. Chem. C* **122**, 22849-22855, (2018).
4. Streiffer, S. *et al.* Domain patterns in epitaxial rhombohedral ferroelectric films. I. Geometry and experiments. *J. Appl. Phys.* **83**, 2742-2753, (1998).
5. Tagantsev, A. K., Cross, L. E. & Fousek, J. *Domains in ferroic crystals and thin films*. (Springer, New York, 2010).
6. Lee, C., Wei, X., Kysar, J. W. & Hone, J. Measurement of the elastic properties and intrinsic strength of monolayer graphene. *Science* **321**, 385-388, (2008).
7. Bertolazzi, S., Brivio, J. & Kis, A. Stretching and breaking of ultrathin MoS_2. *ACS Nano* **5**, 9703-9709, (2011).
8. Falin, A. *et al.* Mechanical properties of atomically thin boron nitride and the role of interlayer interactions. *Nat. Commun.* **8**, 15815, (2017).
9. Tu, Q. *et al.* Stretching and breaking of ultrathin 2D hybrid organic-inorganic perovskites. *ACS Nano* **12**, 10347-10354, (2018).
10. Komaragiri, U., Begley, M. & Simmonds, J. The mechanical response of freestanding circular elastic films under point and pressure loads. *J. Appl. Mech.* **72**, 203-212, (2005).
11. Lv, S.-J., Yin, G.-X., Cui, H.-L. & Wang, H.-Y. A predicted non-layered phase of In_2Se_3 by first principles. *Solid State Commun.* **325**, 114159, (2021).
12. Li, X. *et al.* Exotic magnetism in As-doped α/β-In_2Se_3 monolayers with tunable anisotropic carrier mobility. *Phys. Chem. Chem. Phys.* **21**, 19234-19241, (2019).
13. Aizu, K. Determination of the state parameters and formulation of spontaneous strain for ferroelastics. *J. Phys. Soc. Jpn.* **28**, 706-716, (1970).
14. Aizu, K. Possible species of "ferroelastic" crystals and of simultaneously ferroelectric and ferroelastic crystals. *J. Phys. Soc. Jpn.* **27**, 387-396, (1969).
15. Sader, J. E. Frequency response of cantilever beams immersed in viscous fluids with applications to the atomic force microscope. *J. Appl. Phys.* **84**, 64-76, (1998).
16. Soergel, E. Piezoresponse force microscopy (PFM). *J. Phys. D: Appl. Phys.* **44**, 464003, (2011).
17. Chen, Q. N., Ou, Y., Ma, F. & Li, J. Mechanisms of electromechanical coupling in strain based scanning probe microscopy. *Appl. Phys. Lett.* **104**, 242907, (2014).
18. Kim, Y. *et al.* Nonlinear phenomena in multiferroic nanocapacitors: Joule heating and electromechanical effects. *ACS Nano* **5**, 9104-9112, (2011).
